# Sex differences in fetal intracranial volumes assessed by in utero MR imaging

**DOI:** 10.1186/s13293-023-00497-9

**Published:** 2023-03-15

**Authors:** Paul D. Griffiths, Deborah Jarvis, Cara Mooney, Michael J. Campbell

**Affiliations:** 1 Academic Radiology, University of Sheffield, Sheffield, UK; 2 Clinical Trials Research Unit, School of Health and Related Research, University of Sheffield, Sheffield, UK; 3 Medical Statistics Group, School of Health and Related Research, University of Sheffield, Sheffield, UK

**Keywords:** In utero MR imaging, Brain volume, Gender differences

## Abstract

**Background:**

The primary aim of the study is to test the null hypothesis that there are no statistically significant differences in intracranial volumes between male and female fetuses. Furthermore, we have studied the symmetry of the cerebral hemispheres in the cohort of low-risk fetuses.

**Methods:**

200 normal fetuses between 18 and 37 gestational weeks (gw) were included in the cohort and all had in utero MR, consisting of routine and 3D-volume imaging. The surfaces of the cerebral ventricles, brain and internal table of the skull were outlined manually and volume measurements were obtained of ventricles (VV), brain parenchyma (BPV), extraaxial CSF spaces (EAV) and the total intracranial volume (TICV). The changes in those values were studied over the gestational range, along with potential gender differences and asymmetries of the cerebral hemispheres.

**Results:**

BPV and VV increased steadily from 18 to 37 gestational weeks, and as a result TICV also increased steadily over that period. TICV and BPV increased at a statistically significantly greater rate in male relative to female fetuses after 24gw. The greater VV in male fetuses was apparent earlier, but the rate of increase was similar for male and female fetuses. There was no difference between the genders in the left and right hemispherical volumes, and they remained symmetrical over the age range measured.

**Conclusions:**

We have described the growth of the major intracranial compartments in fetuses between 18 and 37gw. We have shown a number of statistically different features between male and female fetuses, but we have not detected any asymmetry in volumes of the fetal cerebral hemispheres.

## Highlights

- In utero magnetic resonance imaging can be used to calculate intracranial volumes in fetuses.
- In this paper, we present volume data of the major intracranial compartments (cerebral ventricular system (VV), brain parenchymal volume (BPV), extraaxial CSF volume (EAV) and total intracranial volume (TICV) in normal male and female fetuses over a wide gestational age.
- We have shown that BPV and TICV increase at a statistically significant greater rate in male fetuses when compared with female fetuses.
- As a result, male fetuses have statistically significant larger BPV and TICV than female fetuses after 24 gestational weeks.
- There was no statistical evidence for asymmetry of hemispheric volumes in neither male nor female fetuses at any gestational age studied.

## Background

In utero magnetic resonance imaging (iuMRI) of the fetal brain is now a widely accepted clinical tool when used as an adjunct to ante-natal ultrasonography because of proven advantages in terms of improved diagnostic accuracy and counselling [1, 2]. Advances in iuMRI technology and post-acquisition data processing now allows calculation of the volumes of the major intracranial compartments of both normal [3–5] and abnormal fetuses [6]. Specifically, it is possible to measure the volume of the cerebral ventricular system (VV), the brain parenchymal volume (BPV), the extraaxial CSF volume (EAV) and, by summation of those three, the total intracranial volume (TICV). Post-acquisition data processing using the same methodology can also be used to produce surface representations of those compartments.

In this study, we have used iuMRI to measure the intracranial compartmental volumes of a large cohort of normal fetuses over a wide gestational range in the second and third trimesters. This allows us to study changes in intracranial volumes during the course of pregnancy and the primary aim of the study is to test the null hypothesis that there are no statistically significant differences in intracranial volumes between male and female fetuses. We also evaluate the alternative hypothesis that male fetuses have larger intracranial volumes and if so, when do the differences become apparent. Furthermore, we take the opportunity to study the symmetry of the cerebral hemispheres in the cohort, looking for possible asymmetry related to maturity and sex of the fetuses.

## Methods

### Participants

All the pregnant women whose fetuses are reported in this paper were recruited into the MERIDIAN study (magnetic resonance imaging to enhance the diagnosis of fetal developmental brain abnormalities in utero) [1, 2]. Specifically, they were part of an additional study to examine iuMRI scans of brains of fetuses considered to be normal on ultrasound [7]. Ethical approval was obtained from Yorkshire and the Humber/South Yorkshire ethics committee (11-YH-0006) and each woman provided fully informed, written consent. Fetuses are considered, a priori*,* to be normal because they were from a low-risk pregnancy, had no abnormalities on ante-natal ultrasonography (brain or somatic) and had normal brains on iuMR imaging. A total of 200 women with singleton pregnancies between 18 and 37 gestational weeks (gw) were scanned. As reported previously, two of the original 200 fetuses had unexpected brain abnormalities on iuMR [7] and for the purpose of this study, they were replaced by two further normal fetuses with gestational ages matched to the two fetuses with abnormalities. Some results of volumetric analyses on the cohort have been published previously in order to describe normative data by gestational age. However, that analysis did not investigate the effect of the sex of the fetus [5].

### MRI data acquisition and processing

The iuMRI protocol has been reported in full elsewhere [7], but is summarised here. All pregnant women were scanned on the same 1.5-T whole body scanner (Signa HDx, GE Healthcare, Milwaukee) at the University of Sheffield’s magnetic resonance facilities. Routine iuMRI of the fetal brain imaging consisted of T2-weighted single-shot fast spin echo sequences in the three natural orthogonal planes, and T1- and diffusion-weighted imaging both in the axial plane only. Those imaging studies were used to confirm normality of the brain following review by a pediatric neuroradiologist with over 18 years’ experience of fetal neuroimaging (PDG). In addition, volumetric brain imaging was acquired using a balanced steady-state imaging sequence (Fast Imaging Employing Steady-state Imaging—FIESTA, GE Healthcare, Milwaukee) in the axial plane. Those datasets were processed by a senior research MR radiographer with over 8 years’ experience of the technique (DJ) using ‘3D Slicer’ software (www.slicer.org). The surfaces of the cerebral ventricles, brain and internal table of the skull were outlined manually and absolute volume measurements were obtained by multiplying the number of voxels by the voxel size in each segmented compartment. This allowed the direct measurement of VV, BPV and EAV and TICV was derived by adding VV, BPV and EAV. We have previously described good intra- and inter-observer reproducibility of this technique [3, 4] (see discussion). The investigators did not know the sex of the fetus at the time the volume measurements were made as that information was collected post-natally, so the measurements were not biased by knowledge of the sex of the fetus.

The final assessments were designed to look at the symmetry of the fetal cerebral hemispheres in terms of volume of the brain parenchyma following division of the BPV datasets into three components, brainstem/cerebellum and two cerebral hemispheres. The brain stem/cerebellum regions were divided from the supratentorial structures on sagittal imaging using an arbitrary, but consistent, construction line extending from the vein of Galen to the posterior clinoid processes. The cerebral hemispheres were then separated using imaging in the axial plane allowing the volumes for the two cerebral hemispheres to be assessed independently. The symmetry of the cerebral hemispheres was assessed with knowledge of which was the left or which was the right hemisphere. This was done by comparing the brain volume data (which only included the fetal head) with the routine 2D imaging in the coronal plane, on which the chest and abdomen are visible. The laterality of the hemispheres determined on the assumption of situs solitus by reference to the position of the thoracic and abdominal organs (left atrium, spleen and stomach on the left side and the liver is on the right side).

### Data analysis

The data were analysed using Stata (Statacorp, College Station, Texas). The volumetric data were plotted against age to examine their relationship using lowess smoothing plots (bandwidth 0.6) to show the shape of the data. The relationship of VV, BPV and TICV with age appeared linear after 24 weeks and so linear regression models were fitted after this age to assess the rate of growth of the brain and to test whether these rates differ by sex of the fetus. Robust standard errors were used because the standard deviations were observed to increase as the mean volumes increase. In order to examine the whole age range, the data were grouped in quarters by gestational age to enable means by age and sex group to be calculated. The data were also log transformed for more detailed statistical analysis of asymmetry of the cerebral hemispheres.

## Results

**Fig. 1:**
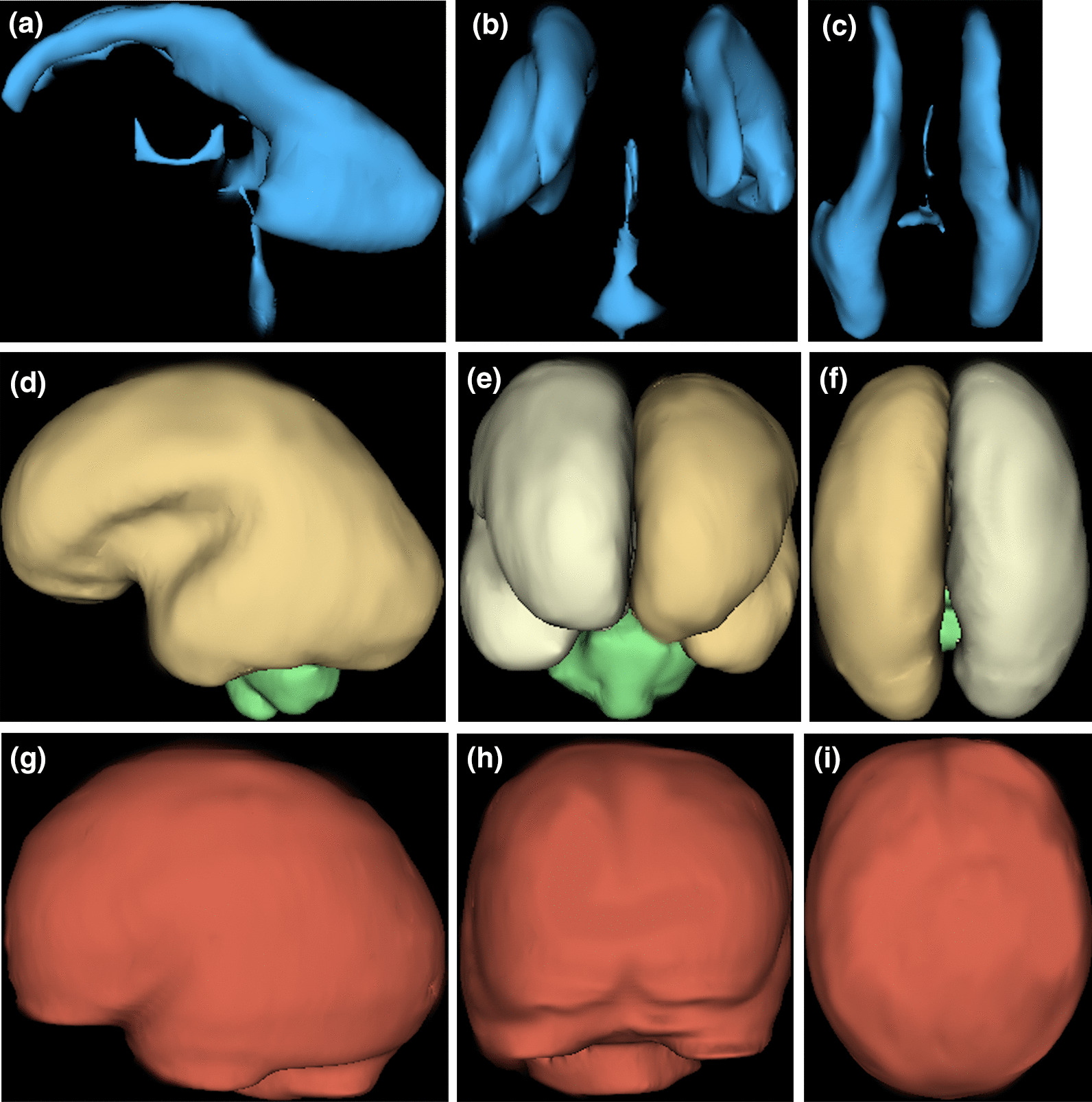
Images of the intracranial compartments studied in this paper in a male fetuses at 22gw. The images are surface representations of the cerebral ventricles (**a** lateral projection, **b** frontal projection and **c** superior projection. The same order is used for the surface representations of the brain parenchyma (**d**–**f**) and the extraaxial CSF spaces (**g**–**i**)

**Fig. 2:**
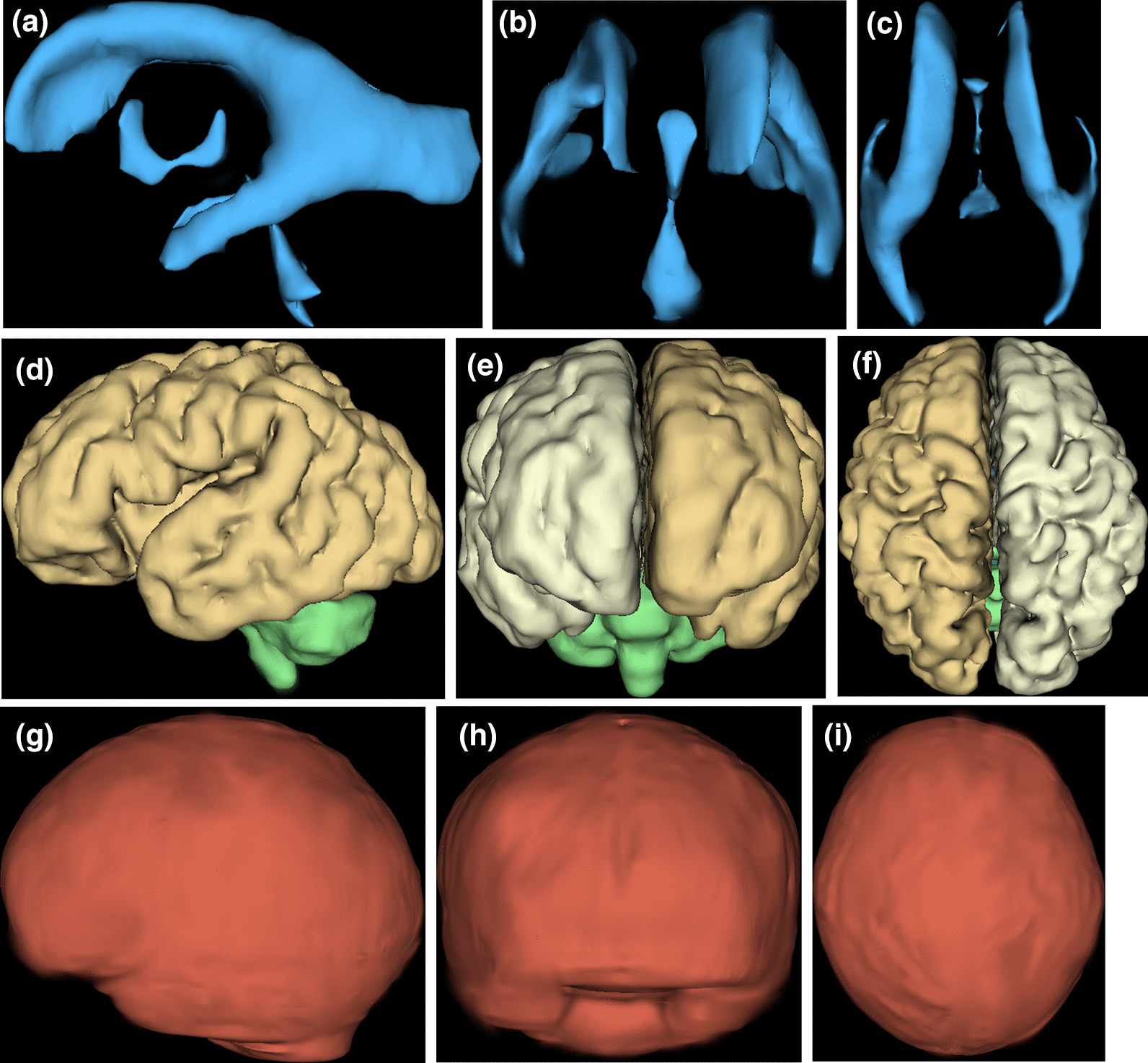
The same format as Fig. 1 for a 34gw male fetus

**Fig. 3:**
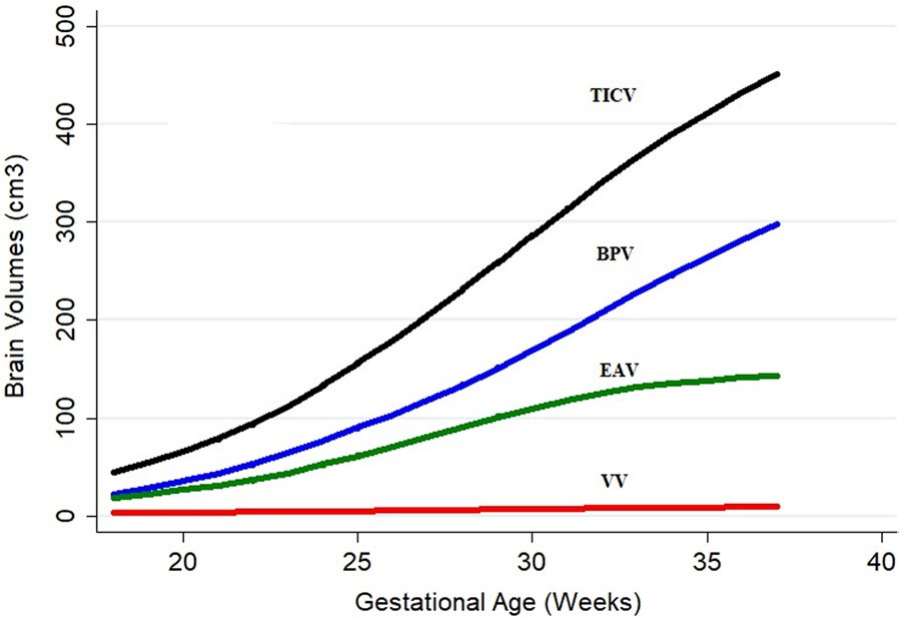
Lowess plots of the four compartmental voulmes studied plotted against gestational age. *VV*  ventricular volume, *EAV* extraaxial volume, *BPV* brain parnchymal volume, *TICV* total intracranial volume

Table 1 shows the distribution of fetuses by age and sex. There were 109 males and 91 females. All age groups from 18 to 37 weeks were represented with a modal age for both males and females of 29 weeks. Representative images of the compartmental volumes at three gestational ages are shown in Figs. 1 and 2 with the lowess smoothing plots for VV, BPV, EAV and TICV plotted against gestational age are shown in Fig. 3. There are different relationships between the volume of the four intracranial compartments and gestational age. The increase in VV is small and close to linear (increasing at approximately 0.3 cm^3^/week) over the study period. The increase in EAV is modest between 23 and 32gw (approximately 10 cm^3^/week) before levelling off after 32gw. The relationship between both BPV and TICV with gestational age is linear after 24gw when the growth rates were approximately 20 cm^3^/week for BPV and 25 cm^3^/week for TICV. Accordingly, the contribution of BPV to the TICV changed substantially over the study period, e.g. BPV accounted for approximately 50% of TICV before 22gw and approximately 65% after 34gw.

**Table 1:** Distribution of fetuses in the study by age and sex

Gestational age (wks)	*n* Female	*n* Male	Total
18–23	22	33	55
24–27	26	22	48
28–30	28	29	46
31–37	15	25	51
Total	91	109	200

### Analysis of compartmental volumes in relation to sex of the fetus

**Fig. 4:**
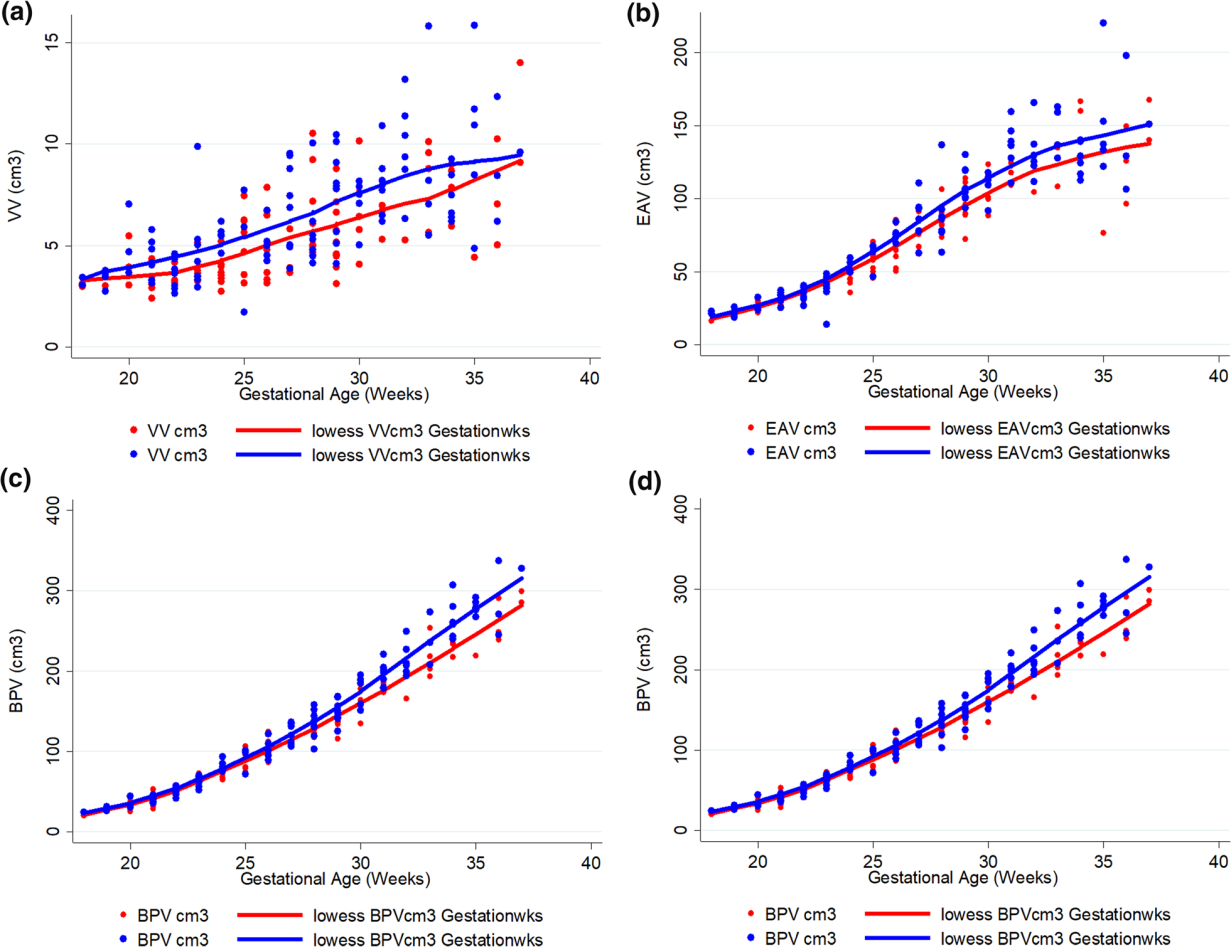
Raw data and smoothing plots for ventricular volume (VV—3a), extraaxial volume (EAV—3b), brain parnchymal volume (BPV—3c) and total intracranial volume (TICV—3d) by gender (males blue, females red)

Figure 4 shows the data plotted by gender in four separate plots with lowess smoothing plots included. Table 2 shows the regression slopes against age and the interaction with sex for the 145 fetuses ≥ 24gw. A number of statistically significant differences between the male and female fetuses are shown. One of the main differences in terms of the slopes between male and female fetuses is for BPV with a greater rate of growth for males of 3.15 cm^3^/week (95% CI 1.27 to 5.02). Because BPV is the major contributor to TICV in more mature fetuses, there is a similar pattern of difference in slopes for TICV, which is also statistically significant. There was no statistically significant differences in growth rates between males and females for VV and EAV. The absolute volumes of male and female fetuses (as opposed to growth rates) were studied after the data grouped into quarters by gestational age as shown in Table 3. There is little difference in BPV and TICV between the sexes until 24 weeks, after which male fetuses have statistically significant larger BPV and TICV. For example, after 31gw male fetuses have approximately 7% larger BPV and 8% larger TICV when compared with female fetuses. In contrast, male fetuses have larger VV when compared with females through all of the gestational range studied with the largest difference occurring in the 24–27gw range (approximately 24% larger). The relationship between EAV and gender was not as straightforward, with males having statistically significant larger EAV between 24 and 27gw only.

**Table 2:** Regression slopes in relation to gestational age and the interaction with sex for 145 fetuses imaged ≥ 24gw

	Female		Male		Difference in slopes	*P* value for difference^a^
	Slope	SE	Slope	SE		
VV	0.37	0.08	0.39	0.08	0.02	0.84
EAV	7.70	0.76	8.12	0.82	0.42	0.71
BPV	15.7	0.58	18.9	0.75	3.2	0.001*
TICV	23.8	1.10	27.4	112	3.6	0.024*

*VV* ventricular volume*, EAV* extraaxial volume*, BPV* brain parnchymal volume*, TICV* total intracranial volume

^*^Slopes statistically significant (*P* < 0.001)

^a^Using standard errors robust to variance heterogeneity

**Table 3:** Absolute volumes of the four intracranial compartments in male and female fetuses after the data were grouped into quarters by gestational age

Variable (cm^3^)	Gestational age (wks)	Female		Male		Male–female	*P*
		Mean	SD	Mean	SD	Mean	
VV	18–23	3.54	0.76	4.14	1.40	0.60	0.070
	24–27	4.61	1.47	5.88	1.87	1.27	0.011
	28–30	6.16	2.01	6.82	2.00	0.66	0.27
	31–37	7.70	2.36	8.91	2.75	1.21	0.12
EAV	18–23	31.8	8.46	31.2	8.53	− 0.6	0.79
	24–27	60.2	14.5	73.6	17.2	13.4	0.005
	28–30	94.5	12.5	102.4	17.8	7.9	0.088
	31–37	128.7	23.5	138.8	24.0	10.1	0.15
BPV	18–23	44.1	14.3	44.1	13.2	0.07	0.98
	24–27	91.6	19.3	104.0	20.0	12.4	0.034
	28–30	140.4	15.5	150.2	22.2	9.8	0.087
	31–37	236.1	36.4	256.2	38.1	18.1	0.12
TICV	18–23	79.4	22.5	79.5	20.7	0.1	0.99
	24–27	156.4	32.4	183.5	37.0	27.1	0.010
	28–30	241.0	23.1	259.4	35.0	18.3	0.040
	31–37	360.7	54.8	390.8	52.2	30.1	0.056

*VV*  ventricular volume, *EAV*  extraaxial volume, *BPV*  brain parnchymal volume, *TICV*  total intracranial volume

### Symmetry of the cerebral hemispheres

**Fig. 5:**
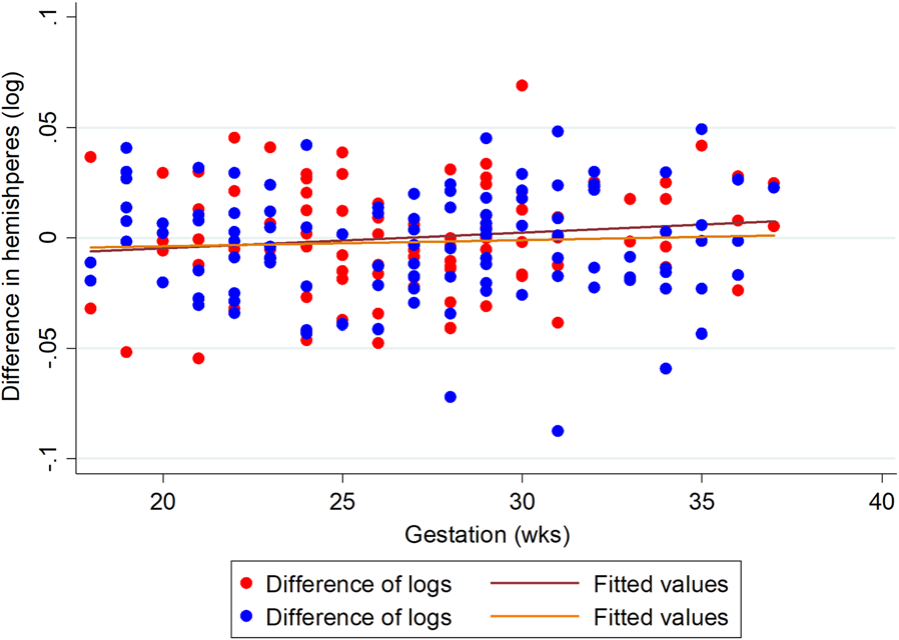
Difference in log hemispheric brain volumes (right–left) by gender (blue male, red female)

The difference between left and right hemispheres is shown in Fig. 5 with the regression slopes (the log of the data is shown as this stabilises the variance). The mean maximum asymmetry between the hemispheres is reasonably constant across the gestational range studied (approximately 2%). The largest asymmetry between the cerebral hemispheres in a fetus was 8.8% and there were only three fetuses with asymmetries > 6%. Accordingly, there is no statistical evidence that the volumes of the fetal cerebral hemispheres differ in size (mean − 0.0019, 95% CI − 0.0053 to 0.0015) *p* = 0.27). There is no statistical evidence to suspect the differences in hemispheric volumes is affected by gender (difference boys minus girls = 0.0038978, 95% CI − 0.0031 to 0.011, *p* = 0.28) or gestational age (*r* = 0.056, *p* = 0.332). In addition, the standard deviations of the differences of the logged data were very similar (0.024 for female fetuses and 0.025 for male fetuses).

## Discussion

In this paper, we have studied volumes of the intracranial compartments of normal fetuses between 18 and 37gw, a period when there is considerable growth and maturation of the fetal brain. Intracranial compartmental anatomy is a complex subject and our approach in this study has involved considerable simplification because of the limitations of iuMRI in its present form in terms of anatomical and contrast resolution. However, the heavily T2-weighted images produced by the 3D acquisition used in this study allow good delineation of the major intracranial compartments because of the favourable contrast differences between the brain, skull and CSF. VV measured in this study also includes the non-ventricular fluid-containing structures (primarily the cavum septum pellucidum and cavum vergae) and the CSF-producing structures (choroid plexi). As well as CSF the EAV contains the majority of the intracranial vascular compartment (large/medium arteries, large cerebral veins and venous sinuses) as they cannot be delineated from the CSF using current imaging methods.

It is important to restate that three compartmental volumes were measured directly in this study (VV, BPV and EAV), whereas TICV was derived from summation of the three compartmental volumes. The technique we have used relies on manual segmentation of the three intracranial compartments and we have previously shown high reproducibility of the method [3, 4], as shown by a mean inter-observer difference of 1.27% (standard deviation ± 4.8%, full range 0.05 to 9.31%). The ‘compartmental’ approach in assessing the intracranial contents of the fetus we describe in this paper is not used in routine clinical practice at present using either ultrasonography or MR imaging, primarily because of the technical challenges in obtaining and processing the data. Instead, linear measurements of the fetal skull are taken by way of head circumference, bi-parietal diameter and/or occipito-frontal diameter. An assumption is made that, in some way, increasing skull size reflects brain growth. This may be true in many cases, but there are relatively common fetal neuropathologies that will interfere with that relationship as discussed later in this section.

With the appropriate technology in place, we aimed to study a large cohort of 200 normal fetuses between 18 and 37gw and measure the intracranial compartmental volumes in order to comment on the normal pattern of growth, sex difference and asymmetry of the cerebral hemispheres. We have shown that there is a close association between brain growth (as indicated by TBV) and head/calvarial growth (as indicated by TICV), particularly after 24gw when the growth trajectories for both compartments are linear and virtually parallel. The close association between BPV and TICV is supportive of the thesis that growth of the brain stimulates mesenchymal development and growth of the skull particularly the bones that develop by intramembranous ossification. The BPV/TICV ratio increases over the gestational range studied because the increase in the volume of the CSF spaces (VV and EAV) were modest in comparison to BPV and TICV. In particular, VV only shows a minor increase in volume whereas the growth of the EAV is modest and plateaus after 32gw. We also had the opportunity to evaluate potential difference in compartmental volumes in relation to the sex of the fetus. Again, BPV and ICV show very similar changes over the time period studied in both sexes but there are differences between male and female fetuses. Although BPV and TICV are very similar in male and female fetuses between 18 and 23gw, male fetuses subsequently grow at rates that are statistically significantly larger (by 7–8%) when compared with female fetuses.

As already stated, clinical assessment of skull size uses linear rather than volume measurements and it should be appreciated that an 8% difference in TICV is commensurate with a 2.6% difference in linear dimensions only. Ultrasound-derived biometric charts of normal fetuses used in clinical practice do not distinguish between male and female fetuses (e.g. intergrowth 21.tghn.org/fetal-growth (based on Papageorghiou et al. [8]), but biometric charts of babies born prematurely do (e.g. intergrowth21.ndog.ox.ac.uk). Analysis of those charts shows small differences between male and female babies although males are consistently larger, e.g. head circumference median (standard deviation) at 32gw males 29.4 cm (± 1.6 cm), females 29.1 cm (± 1.6 cm); at 36gw males 32.5 cm (± 1.3 cm), females 32.1 cm (± 1.2 cm). It is also widely accepted that boys are slightly larger than girls following delivery at Term, hence the use of gender specific normative charts post-natally. An important recent paper by Galjaard et al. has studied head size in relation to the sex of the fetus [9]. Those authors reported nearly 28,000 fetuses from a low-risk Caucasian population and they showed that male fetuses have larger bi-parietal diameters and head circumferences when compared with female fetuses from 20gw. As predicted by the results of our study, the differences were small (amounting to a 3-day difference between 20−24gw) but were statistically significant at the *p* > 0.001 level.

Another interesting and potentially clinically relevant finding is found in the gender difference in VV, which were larger in male fetuses (ranging from 11 to 17% bigger through the gestational range). In a comparable fashion to the TICV discussion, those differences equate to only a 5% difference in linear measurements of the ventricles, such as the width of the trigone of the lateral ventricles that is used in routine assessments of the fetus. A fetus is considered to have ventriculomegaly if the trigone measurement is > 10 mm at any stage of pregnancy on the basis that 10 mm is approximately 4sd above the mean value [10]. Between 1 and 3/1000 of unselected fetuses have trigone measurements ≥ 10 mm, described as ventriculomegaly in clinical practice. The results of the present study show two important caveats when using the accepted current dogma about trigone size. First, VV normally increases with gestational age and although the absolute changes are small there is a more than doubling in VV between 20 and 36gw. It may not be appropriate, therefore to have the same 10-mm cut-off for all gestational ages. Secondly, the statistically significant larger VV in normal male fetuses is likely to explain the consistent observation of an excess of male fetuses diagnosed with isolated ventriculomegaly, which ranges from 1.3:1 to 3.5:1 [11–14].

We also investigated the symmetry of the fetal cerebral hemispheres. There are many descriptions of asymmetry of the mature human cerebral hemispheres and those are generally considered to be important because of functional localisation of the cerebral hemispheres and cerebral ‘dominance’, particularly for language. In children and adults, it is estimated that approximately 90% of right-handed people are left hemisphere dominant on the basis of language localisation and slightly fewer left-handed people are left hemisphere dominant. However, in spite of marked ‘functional asymmetries’ the mature cerebral hemispheres do not show major structural asymmetry. In fact, the only relatively consistent asymmetries in the post-natal human cerebral hemispheres are in the superior temporal lobes and ‘Yakovlevian anti-clockwise torque [15]’. The planum temporale, on the posterior aspect of the superior temporal lobe is involved in language processing and is usually larger in right-handed people. Yakovlevian anti-clockwise torque refers to an apparent twisting of the cerebral hemispheres (anti-clockwise if looking at the brain from above—see Le May 1976 [16]) so that right frontal lobe extends across the midline to the left and the left occipital lobe extends across the midline posteriorly. We have not studied whether this feature is present in fetuses formally in this paper but our initial impression that it is not. In spite of that arrangement, the human mature cerebral hemispheres are not considered to be different in terms of weight or volume [15]. Similarly, there was no evidence of asymmetry in volume of the human fetal cerebral hemispheres in our present study or, more accurately, no asymmetry could be detected in relation to the precision of technique (with an inter-observer error of 1.27%). This is true for the entire cohort and when the cohort was divided in terms of gender. We have not studied differences in sulcation/gyration patterns in our cohort, but a previous iuMR study showed more advanced sulcation in the left temporal lobe when compared with the right [17].

It is important to consider the rationale for obtaining normal values for the volumes of the intracranial compartments in second and third trimester fetuses. Some insight can be gained by considering the expected changes in compartmental volumes in some of the commoner causes of fetal ventriculomegaly. For example, ventriculomegaly is the commonest abnormal intracranial finding on ante-natal ultrasonography and hence the commonest referral for iuMR neuroimaging. In most cases fetal ventriculomegaly is an isolated finding, and such fetuses have very good prospects of a normal neurodevelopmental outcome. In those fetuses the expected pattern of intracranial volumes change would be—increased VV but BPV, EAV and TICV within normal ranges. Alternatively, if fetal ventriculomegaly is secondary to non-communicating hydrocephalus (due to a blockage in CSF flow in the ventricles) the VV will be increased (often massively) and the associated raised intraventricular pressure will cause effacement of the extraaxial CSF spaces (hence reduced EAV). The bones of the calvarium are not fused in the fetus, so the raised intraventricular/intracranial pressure will also cause increased TICV, whilst BPV will often be normal or slight reduced. In contrast, if ventriculomegaly is due to a destructive process of the brain (such as the result of trans-placental viral infection or in utero hypoxic ischaemic injury) VV and EAV are increased by an ex vacuo mechanism secondary to reduced BPV. We have confirmed that brain growth is a major driver for head growth in this paper, therefore reduced TICV should be expected in fetuses with destructive brain pathology. Hence, knowledge of the compartmental volumes can aid diagnosis of fetal neuropathology as well as understanding normal development.

There are several limitations in the current work, first in relation to the cohort size. The size of the sample reported in this paper was determined from the original purpose of the study, namely to determine if fetuses which are normal on ultrasound had any abnormalities on iuMRI. A sample size of 200, in which no abnormalities were observed would mean that the upper 95% limit of the likely rate of abnormalities in fetuses with a normal ultrasound is 1.5%. As such, this activity reported here was not formally powered to show differences in sex. Also, it was not possible to control the content of the cohort in order to have, for example, the same number of cases at each gestational age or to obtain equal numbers of male and female fetuses. We also recognise the potential weaknesses in the technical performance of our methodology in producing the volume measurements. Although we have shown good intra- and inter-observer reproducibility, we cannot compare our measurements with the ‘real’ volumes of the fetal brains, hence we cannot report the accuracy of the technique. In addition, we have described the slopes of our measured volumes in relation to gestational age, which we have implied indicates growth rates of those compartments. Information about fetal growth can only be done formally if repeat measurements are made in the same individual and we stress that there was no capacity to do a longitudinal assessment in our study.

### Perspectives and significance

We have shown that BPV and VV increased steadily over the gestational age 18 to 37 weeks, and as a result TICV also increased steadily over that period. TICV and BPV increased at a statistically significantly greater rate in male relative to female fetuses after 24gw. The greater VV in male fetuses was apparent earlier, but the rate of increase was similar for male and female fetuses. For EAV the mean volumes for males and females appeared to diverge and then converge, resulting in similar linear slopes after 24 weeks. There was no difference between the genders in the left and right hemispherical volumes, and they remained symmetrical over the age range measured.

## Data Availability

The datasets during and/or analysed during the current study are available from the corresponding author on reasonable request.
