# Correction: Sex differences in fetal intracranial volumes assessed by in utero MR imaging

**DOI:** 10.1186/s13293-023-00505-y

**Published:** 2023-04-11

**Authors:** Paul D. Griffiths, Deborah Jarvis, Cara Mooney, Michael J. Campbell

**Affiliations:** 1 Academic Radiology, University of Sheffield, Sheffield, UK; 2 Clinical Trials Research Unit, School of Health and Related Research, University of Sheffield, Sheffield, UK; 3 Medical Statistics Group, School of Health and Related Research, University of Sheffield, Sheffield, UK

**Correction: Biology of Sex Differences (2023) 14:13** 10.1186/s13293-023-00497-9

Following publication of the original article [1], the authors reported a typesetting error in Fig. 4. Fig. 4c was erroneously duplicated as Fig. 4d.

The correct Fig. 4 is given in this correction article. The original article [1] has been corrected.

**Fig. 4:**
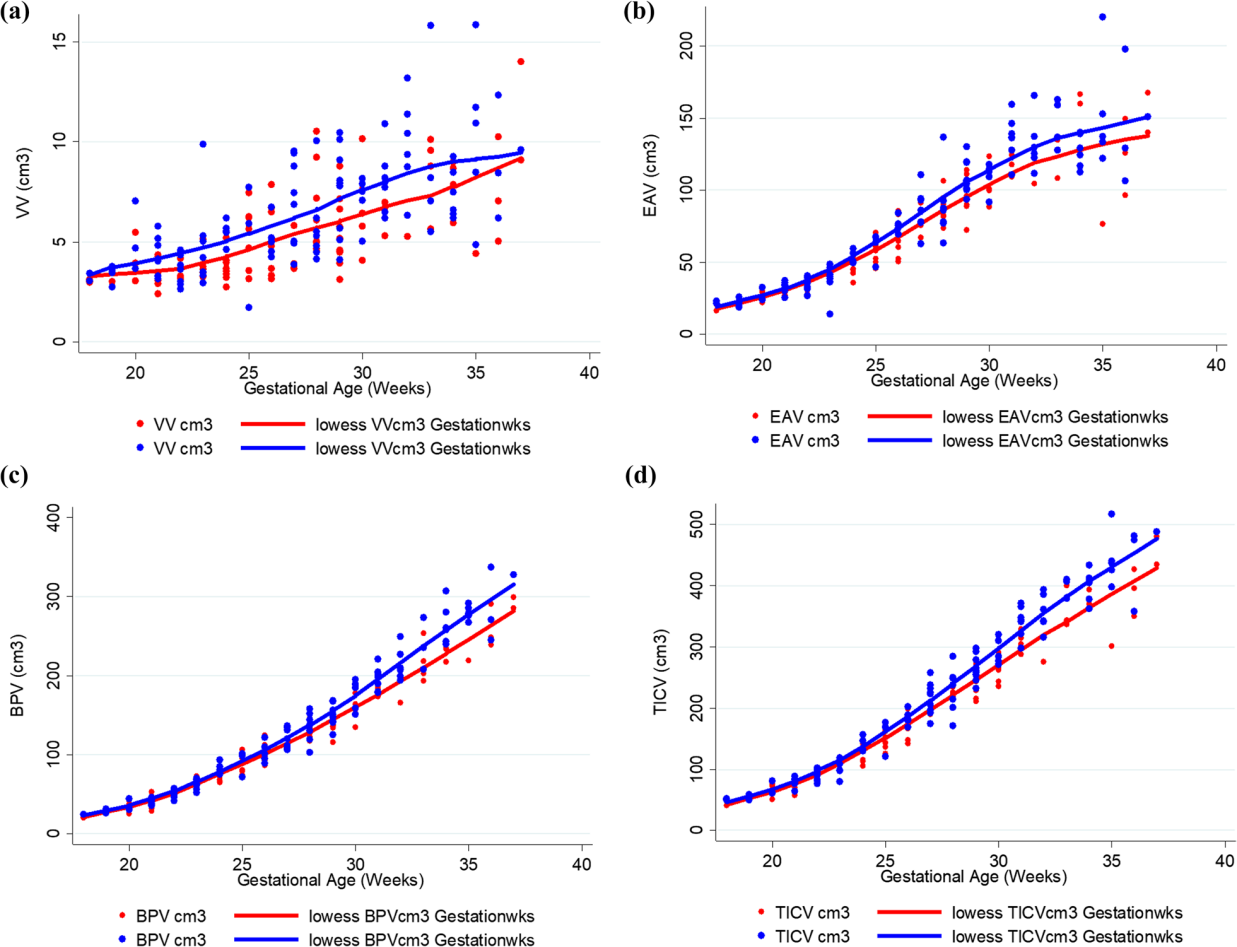
Raw data and smoothing plots for ventricular volume (VV—3a), extraaxial volume (EAV—3b), brain parnchymal volume (BPV—3c) and total intracranial volume (TICV—3d) by gender (males blue, females red)
